# Clinical features and metabolic complications for non-alcoholic fatty liver disease (NAFLD) in youth with obesity

**DOI:** 10.3389/fendo.2023.1062341

**Published:** 2023-01-17

**Authors:** Emiliano Barbieri, Nicola Santoro, Giuseppina Rosaria Umano

**Affiliations:** ^1^ Department of Pediatrics, Federico II University, Napoli, Italy; ^2^ Department of Pediatrics, Kansas University Medical Center, Kansas City, KS, United States; ^3^ Department of Medicine and Health Sciences, “V. Tiberio” University of Molise, Campobasso, Italy; ^4^ Department of Pediatrics, Yale School of Medicine, New Haven, CT, United States; ^5^ Department of the Woman, the Child, and General and Specialized Surgery, University of Campania, Luigi Vanvitelli, Naples, Italy

**Keywords:** NAFLD, type 2 diabetes, youth, insulin resistance, genetics

## Abstract

Pediatric obesity has become in the last forty years the most common metabolic disease in children and adolescents affecting about 25% of the pediatric population in the western world. As obesity worsens, a whole-body insulin resistance (IR) occurs. This phenomenon is more pronounced during adolescence, when youth experience a high degree of insulin resistance due the production of growth hormone. As IR progresses, the blunted control of insulin on adipose tissue lipolysis causes an increased flux of fatty acids with FFA deposition in ectopic tissues and organs such as the liver, leading to the development of NAFLD. In this brief review, we will discuss the clinical implications of IR and NAFLD in the context of pediatric obesity. We will review the pathogenesis and the link between these two entities, the major pathophysiologic underpinnings, including the role of genetics and metagenomics, how these two entities lead to the development of type 2 diabetes, and which are the therapeutic options for NAFLD in youth.

## 1 Introduction

Nonalcoholic fatty liver (NAFL) is defined as the presence of biopsy-proven fat accumulation in more than 5% of hepatocytes without other causes of intrahepatic fat accumulation (i.e., excessive alcohol assumption, infectious diseases, autoimmunity, and metabolic diseases) (1). The gold standard for diagnosis is liver biopsy, as it allows a more accurate quantitative assessment of hepatocyte fat content. However, in clinical practice, less invasive methods, such as magnetic resonance imaging and liver ultrasound, are performed in pediatric age (1). Nonalcoholic fatty liver disease (NAFLD) refers to a variety of clinicopathological entities, ranging from simple hepatic steatosis (NAFL) to steatohepatitis (NASH), cirrhosis, and end-stage liver disease even at a young age (1). Children and adolescents with overweight and obesity present an increased risk for developing NAFLD early on, with higher rates in male adolescents. Moreover, NAFLD is characterized by ethnic differences, with Hispanic youth showing higher rates than - Non-Hispanic White (NHW) and Non-Hispanic Black (NHB), and the latter group having the lowest prevalence even when severe obesity is present (2).

Early autoptic reports (3) in 742 youth show that in the general pediatric population between age 2 and 19 years the prevalence of NAFLD is about 9.6%, and is the lowest (0.7%) between 2 and 4 years of age and the highest (17.3%) between 15 and 19 years of age (3). NAFLD prevalence increases with the increase of the degree of adiposity, being about 38% in youngsters with obesity (2–4). NAFLD prevalence changes among different ethnicities/races being about 40% among Hispanics, 13% in Non-Hispanic Black (NHB) and 30% in Non-Hispanic White (NHW) (4, 5). Data from Trico’ et al. show that in NHB individuals with diagnosis of NAFLD show the same degree of disease as NHW and H (2), and a more pronounced degree of insulin resistance as well as higher prevalence of prediabetes and type 2 diabetes (2). The reason for these ethnic/race differences is unknown and may be due to differences in metabolic pathways involved in the pathogenesis of NAFLD (such lipogenesis, adipose tissue lipolysis etc.) or to differences in adipose tissue distribution. Differences in the risk of developing NAFLD exist also between boys and girls, with girls showing a lower risk (3, 5), probably due to the estrogens, that seem to have a protective effect against intrahepatic fat accumulation (6, 7).

There are very few studies assessing the natural history of pediatric onset NAFLD and most of them are retrospective. Despite that, all show that early onset NAFLD has deleterious long-term effects. A landmark retrospective study by Feldstein et al. carried out reviewing the charts of 66 youth (average age 13.9+/-3.9 years) showed that youth with early onset NAFLD have standardized mortality ratio of 13.6 during the second decade of life compared to the general population of same age and gender (8). The long-term effects of NAFLD also affect insulin action worsening insulin resistance occurring in youth with obesity (9).

## 2 Methods

We performed a literature search in the PubMed database including the terms: “NAFLD”, “children and adolescents”, “insulin-resistance”, “type 2 diabetes mellitus”. These terms were used in different combinations. Only English language papers were included. Articles were evaluated for scientific relevance and pertinence to the topic. Relevant references from selected papers were included.

## 3 Pathogenesis of NAFLD

Hepatic fat accumulation represents the main feature of NAFLD (1). Triglyceride’s accumulation in the hepatocyte results from the excessive flux of adipose tissue-derived free fatty acids (FFA), enhanced hepatic *de novo* lipogenesis, chylomicron remnants accumulation, and impaired hepatic beta-oxidation (10, 11). The resulting imbalance between FFA intrahepatic concentration and esterification with glycerol to form triglycerides promotes intrahepatic fat deposition and IR. Not only quantitative fat deposition, but also qualitative lipid composition is a relevant determinant of liver injury progression and NAFLD-related metabolic comorbidities occurrence (12, 13). In fact, excess of omega-6 polyunsaturated fatty acids have been sown to be detrimental for the liver health (14, 15).

Along with nutritional factors, the role of genetic, microbial, metabolic, and other environmental factors has been investigated (16–19). Several common genetic variants have been associated with NAFLD in adults and youth. The I148M variant in the *PNPLA3* gene is the most important genetic determinant of NAFLD (20). It interacts with nutrients, insulin resistance, and visceral adiposity (21–24) to convey susceptibility to the disease. The *PNPLA3* gene encodes for the adiponutrin, a protein expressed in the adipose tissue and the liver with a strong lipolytic activity on phospholipids (20). The I148M substitution causes a loss-of-function with impaired lipase activity, hepatic fat accumulation, and macrovescicular steatosis (25, 26). This variant displays a geographical distribution heterogeneity, being less frequent in individuals of African descents (27). This finding might explain the lower prevalence/incidence of NAFLD in this ethnic group even in presence of severe obesity and IR (27, 28). Another relevant genetic variant is the E167K allele in the Transmembrane 6 superfamily member 2 (*TM6SF2*) gene (29). TMS6F2 is a 7-domain transmembrane transporter involved in VLDL secretion from the liver (29). When the aminoacid change occurs, the VLDL secretion is impaired leading to hepatic fat accumulation and at the same time conferring a lower cardiovascular risk, given to the lower concentrations of pro-atherogenic lipoproteins (30, 31). The P446L variant in the *GCKR* gene is another recognized common genetic variant associated with NAFLD (32). The *GCKR* encodes for the GCKRP (glucokinase regulatory protein) that binds the glucokinase (GCK) in the nucleus of the hepatocytes. When the *GCKR* the P446L occurs, the mutated protein shows an impaired ability to bind the GK, the consequence being that more GK is available in the cytoplasm to convert the glucose in glucose-6-phosphate, its active form (33). This leads to an enhanced glycolysis and hepatic *de novo* lipogenesis (34). Other variants have been reported in *MBOAT7* (35, 36) and *HSD17B13* (37, 38) genes with minor effect size, but interestingly enough only the variant in the *MBOAT7* gene has been associated also with IR in youth (35).

Gut-liver axis is another important player in the pathogenesis of NAFLD and its progression. The liver may affect the composition of gut microbiota *via* the bile acids and the secretion of antimicrobial compounds in the intestine (39, 40). At the same time, the gut microbiota is involved in nutrient absorption and gut permeability. Therefore, gut dysbiosis might induce hepatic fat deposition and inflammatory insults to the liver. It has been shown that an altered gut microbiota composition and reduced microbiota variety in subjects with NAFLD compared to those without NAFLD. A relative prevalence of Gram-negative to Gram-positive bacteria has been described in NAFLD compared to healthy controls, with an increase of Proteobacteria in more severe NAFLD (41). Another study reported a reduction of Bacteroides in pediatric NAFLD compared to non-affected subjects (42). Moreover, lower microbiota α-diversity has been described in pediatric NAFLD (42–45). The microbiota composition is associated with higher expression of genes encoding inflammatory compounds thus leading to more severe forms (43). In a recent shotgun metagenomic study of gut microbiota involving youth with obesity with and without NAFLD, the authors found a higher prevalence of genes coding for branched chain aminoacids and short chain fatty acids in the group with NAFLD (46).

## 4 Mechanisms underlying the association between NAFLD and IR

NAFLD is commonly associated with other metabolic comorbidities including dyslipidemia, and type 2 diabetes mellitus (T2D), with insulin resistance (IR) being the main pathogenic link between these conditions (2, 8). A bidirectional effect between NAFLD and IR has been postulated. In fact, IR is a predictor of NAFLD development in youth, and patients with T2D display higher rates of NAFL, nonalcoholic steatohepatitis (NASH), and advanced fibrosis compared to non-diabetic subjects (47). It has been estimated that about 30% of children with NAFLD might present with prediabetes or T2D (47). Studies investigating IR have demonstrated that NAFL per se is a risk factor for IR independent of the effect of other ectopic fat depots (i.e., visceral adipose tissue and intramyocellular fat depots (48). A study focusing on youth with obesity and NAFLD reported also a decrease in beta-cell function paralleling with hepatic steatosis severity degree (49). The association between NAFLD and IR is mediated by intracellular compounds, namely diacylglycerols (DAG) and ceramides that affect insulin transcription. Early studies by the Shulman lab at Yale University have shown in mice and humans that DAG accumulation impact insulin action in the liver, muscle and adipose tissue, the consequence being higher fasting glucose availability in tissues and plasma (50, 51). In fact, adipose tissue insulin resistance will result in an increased adipose tissue lipolysis with increased glycerol flux to the liver, where it serves as substrate for the gluconeogenesis (accounting for about 40% of neo-gluconeogenesis) (51). IR in the liver will result in an increased glucose production and in the muscle in a reduce glucose uptake. Overall, the increased availability of FFA and glucose in the liver will provide more substrate for the Acetyl-CoA formation and for the hepatic *de novo* lipogenesis, a pathway enhanced in the context of IR (52). From a clinical standpoint the consequence is a higher risk of T2D over time.

## 5 Therapeutic options for NAFLD in youth

The discovery of GLP-1 has resulted in the development of GLP-1 analogues, initially used to treat T2D in adults and youth and then approved also for the treatment of obesity as they have a strong anorexigenic effect. However, despite evidence showing an effect of semaglutide (53)on NAFLD, this class of medications is not approved for NAFLD, but for obesity (54) and type 2 diabetes (55) in youth. Even though the GLP-1 analogues are indeed game changer in the therapy of obesity and its complications, lifestyle intervention represent the first line of treatment. Nutritional approaches are, in fact, effective in ameliorating NAFDL and IR. A study testing the effect of 8-week low sugar diet (56) and a study testing a low omega 6 to omega 3 PUFA ratio for 12 weeks (12) showed that these nutritional interventions reduced MRI assessed intrahepatic fat of about 30% and had a similar effect on IR. Western diet is characterized by excess of free sugars and imbalance of high omega 6 and low omega 3 PUFA. The increased hepatic uptake of omega 6 PUFA leads to oxidized linoleic acid metabolites production and finally fatty acids accumulation (57, 58). Therefore, modification of dietary fat intake play an important role in NAFLD treatment. In addition, increased intake of certain dietary sugar, in particular fructose, have been associated with increased risk of NAFLD. Fructose is highly lipogenic and is added in processed foods and beverages (59). Low sugar diets are a promising tool for NAFLD therapy. Finally, preclinical studies have suggested that gut microbiome modulation might represent a therapeutic option for NAFLD. Several clinical studies have reported that NAFLD is associated with less microbiome diversity and relative prevalence of Gram-negative bacteria compared to non-NAFLD subjects (60). More recently, systematic review and metanalysis have investigated the effectiveness of probiotics in the treatment of NAFLD. The available RCT are highly heterogeneous in terms of probiotics supplementation, outcome measures, and diagnostic tools for NAFLD. Therefore, even if a decrease in liver enzymes and NAFLD severity has been reported, there is no sufficient evidence to recommend the use of probiotics for NAFLD treatment (60).

## 6 Conclusions

NAFLD is an important complication of childhood obesity strongly linked to IR and T2D in youth. Given the long-term implications that it can have on the mortality and the morbidity of young individuals it is imperative to find new and more effective preventive and therapeutic strategies for this disease.

## Author contributions

EB and GU drafted the manuscript. NS conceptualized the review and reviewed the draft. All authors contributed to the article and approved the submitted version.
